# Comparative Validity of Smartwatch-Derived Heart Rate and Energy Expenditure During Endurance and Resistance Exercise

**DOI:** 10.3390/s26082526

**Published:** 2026-04-19

**Authors:** Tae-Hyung Lee, Dong-Uk Jun, Ju-Yong Bae, Hee-Tae Roh, Su-Youn Cho

**Affiliations:** 1 Exercise Physiology Laboratory, Department of Physical Education, Yonsei University, Seoul 03722, Republic of Korea; 2 Digital Transformation Team, Healthcare/Sensor Business Development, SOLUM, Yongin 16914, Republic of Korea; 3 Department of Physical Education, College of Arts and Sports, Dong-A University, Busan 49315, Republic of Korea; 4 Department of Sports Science, College of Arts and Sports, Sun Moon University, Asan 31460, Republic of Korea

**Keywords:** smartwatch, wearable sensors, photoplethysmography, heart rate monitoring, energy expenditure estimation, validity, exercise modality

## Abstract

Smartwatches are widely used to monitor physiological responses during exercise; however, their accuracy in measuring heart rate (HR) and energy expenditure (EE) across different exercise modalities remains insufficiently characterized. This study evaluated the accuracy of HR and EE measurements obtained from four commercially available smartwatches in comparison with gold-standard reference methods. Sixty-two healthy adult men performed standardized endurance and resistance exercise protocols while simultaneously wearing four smartwatches (Apple, Galaxy, Fitbit, and Garmin). HR was measured using electrocardiography (ECG), and EE was determined using indirect calorimetry. Measurement accuracy was assessed using repeated-measures analysis of variance, Pearson’s correlation analysis, intraclass correlation coefficients (ICCs), and Bland–Altman analyses. All smartwatches demonstrated high accuracy in HR measurements during both endurance and resistance exercises. During endurance exercise, HR measurements from all smartwatch brands were comparable to those obtained via ECG, whereas during resistance exercise, only the Apple Watch showed no significant difference from the ECG. HRs showed strong correlations with ECG readings (*r* = 0.64–0.97), excellent reliability (ICC > 0.94), and narrow limits of agreement (approximately ±10 bpm). In contrast, the EE measurements exhibited limited accuracy across all devices. During endurance exercise, EE was consistently underestimated with wide limits of agreement. EE accuracy further deteriorated during resistance exercise, showing weak correlations with indirect calorimetry (*r* = 0.10–0.34) and poor reliability (ICC < 0.45). Overall, smartwatches provide accurate HR measurements across endurance and resistance exercise modalities, supporting their use in exercise intensity monitoring and HR-based training. However, smartwatch-derived EE estimates do not accurately reflect the metabolic demands, particularly during resistance exercises. Future research should focus on improving EE estimation algorithms through multimodal biosignal integration and machine-learning approaches, and validating these methods across diverse populations and exercise modalities.

## 1. Introduction

According to the Worldwide Fitness Trends survey conducted by the American College of Sports Medicine (ACSM), wearable technology has consistently ranked among the top three global fitness trends since 2016 and was identified as the leading trend in 2025, underscoring its sustained and expanding influence across the fitness and health industries [1]. Wearable technologies include a wide range of devices, such as smartwatches, smart eyewear, and fitness trackers [2]. Among these, smartwatches are most commonly used by consumers to monitor physical activity-related parameters, particularly heart rate (HR) and energy expenditure (EE), as well as to track health-related indicators [3,4,5].

HR monitoring in wearable devices is predominantly based on photoplethysmography (PPG). Since its initial development by Hertzman in 1938 [6], PPG has been widely adopted for noninvasive pulse monitoring in wearable technologies [7]. PPG-based HR measurements rely on detecting changes in light absorption or reflection caused by cyclic variations in blood volume within peripheral tissues. Specifically, light emitted from the PPG sensor penetrates the skin, and fluctuations in the intensity of the reflected or transmitted light, associated with cardiac-induced vascular expansion and contraction, are analyzed to estimate the HR [7,8]. Despite its widespread application, the accuracy of PPG-based HR measurements is limited by several sources of error. Motion-induced artifacts, sensor-skin contact pressure, changes in skin blood flow due to temperature variations, skin pigmentation, and excessive movement at the measurement site have all been reported to degrade the signal quality and reduce the measurement accuracy [6,9,10]. These limitations become particularly relevant during dynamic exercise conditions, in which, movement complexity and mechanical perturbations are amplified.

Inaccuracies in smartwatch-derived HR measurements may further propagate into errors in EE estimation. Several studies have reported the limited accuracy of smartwatch-based EE estimates, particularly during cycling and resistance exercise [11]. Such inaccuracies are likely attributable to the structural limitations of the proprietary algorithms used by wearable devices that infer exercise intensity and metabolic demand based on combinations of HR signals and motion-related data [12,13]. Importantly, while HR represents a directly measured physiological signal, EE is an inferred metric derived through algorithmic modeling, rendering it inherently more sensitive to errors arising from both sensor input and model assumptions. Despite these methodological limitations, the role of wearable technologies in clinical and public health contexts continues to expand, particularly in the management of chronic diseases [14]. HR monitoring is widely used in cardiovascular disease surveillance and management [15], and EE estimation is increasingly being incorporated into interventions for metabolic disorders such as diabetes mellitus [16]. Commercially available smartwatches provide physiological metrics that may be relevant for clinical decision making; however, these values are generated using manufacturer-specific proprietary algorithms. In most cases, the metabolic equations and estimation procedures underlying these algorithms are not disclosed [12,13]. Furthermore, manufacturers rarely provide quantitative indices of measurement accuracy or error rates, limiting the independent evaluation of the clinical reliability of smartwatch-derived HR and EE measurements [13,15].

Previous studies have suggested that smartwatch-based HR measurement can demonstrate acceptable validity under specific conditions, whereas EE estimation exhibits substantial inter-device variability and pronounced errors that depend on the exercise modality and intensity [5,11,12]. However, systematic evaluations that directly compare the validity of HR and EE across distinct exercise modalities, particularly endurance and resistance exercises, within a unified experimental framework remain limited. Therefore, the purpose of this study was to systematically evaluate the validity of smartwatch-derived HR and EE measurements in comparison with reference standard methods. By examining modality-specific accuracy and agreement during endurance and resistance exercises, this study aimed to clarify the conditions under which smartwatch-derived physiological metrics can be reliably interpreted and delineate their limitations for application in clinical contexts, particularly chronic disease management and exercise prescription. Ultimately, through these findings, we aim to inform appropriate interpretation and use of smartwatch-derived physiological data in clinical practice and research settings.

## 2. Methods

### 2.1. Participants

A total of 62 healthy adult men voluntarily participated in this study. The participants had a mean age of 26.55 ± 4.21 years, a mean height of 175.08 ± 5.22 cm, and a mean body mass of 73.29 ± 7.81 kg. The mean body mass index (BMI) was 23.89 ± 2.09 kg/m^2^. Body composition analysis revealed a mean body fat mass of 13.49 ± 4.14 kg and a mean lean body mass of 33.95 ± 3.66 kg.

To minimize potential confounding factors that affect HR responses during exercise, participants were required to have a BMI ≤ 25 kg/m^2^ and to be free from any conditions that could limit exercise participation, including cardiovascular or musculoskeletal disorders. Prior to participation, all individuals received a detailed explanation of the study purpose, experimental procedures, and the potential benefits and risks associated with participation. Written informed consent was obtained from all participants before enrollment. The study protocol was approved by the Institutional Review Board of Yonsei University (IRB No. 202207-HR-2918-02), and all procedures were conducted in accordance with the principles outlined in the latest revision of the Declaration of Helsinki.

### 2.2. Exercise Protocol

Participants completed standardized endurance and resistance exercise protocols. The endurance exercise protocol was designed using the Karvonen formula [16] to calculate individual target HR (THR) zones. Resting HR (HRrest) was measured after at least 10 min of seated rest in a quiet environment prior to the experimental protocol to ensure a stable physiological baseline. Maximal HR (HRmax) was estimated using the age-predicted equation (HRmax = 220 − age) [17]. Exercise intensity was prescribed as a percentage of HR reserve (HRR), calculated as the difference between HRmax and HRrest. The THR was calculated as follows: THR = [(HRmax − HRrest) × exercise intensity(%)] + HRrest.

**Figure 1:**
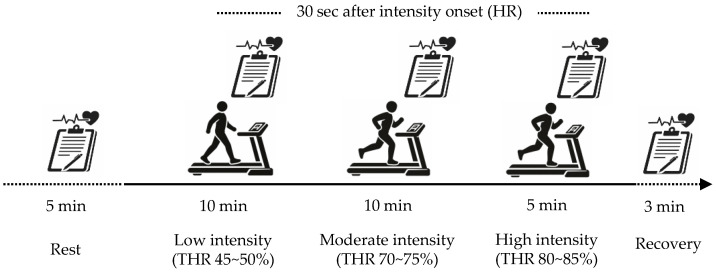
Schematic illustration of the endurance exercise protocol and timing of HR measurements. Participants performed low-intensity (45–50% target heart rate [THR], 10 min), moderate-intensity (70–75% THR, 10 min), and high-intensity (80–85% THR, 5 min) exercise, followed by a 3-min recovery period. HR was recorded 30 s after the onset of each intensity stage.

The protocol consisted of three intensity stages: a low-intensity stage at 45–50% of THR for 10 min, a moderate-intensity stage at 70–75% of THR for 10 min, and a high-intensity stage at 80–85% of THR for 5 min, followed by a 3-min recovery period. The total duration of the endurance exercise protocol was 28 min. To ensure HR stabilization prior to exercise, participants remained seated for 5 min before initiating the endurance exercise, during which, resting HR was recorded. To accurately capture HR responses to changes in exercise intensity, HR values were recorded 30 s after entering each target intensity zone. A standardized warm-up and cool-down, consisting of light walking and stretching for 5 min each, was performed before and after the endurance exercise (Figure 1).

**Figure 2:**
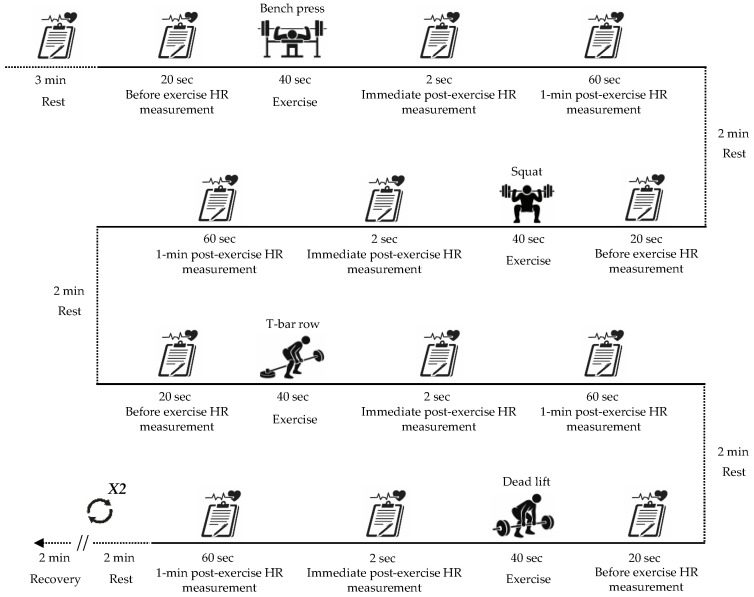
Schematic illustration of the resistance exercise protocol and timing of HR measurements. Participants completed two sets of four resistance exercises (bench press, squat, T-bar row, and deadlift) at a 10-repetition maximum load. HR was measured before exercise, immediately after exercise, and 1 min post-exercise, with standardized rest periods between exercises.

The resistance exercise protocol consisted of two sets of four exercises performed at a 10-repetition maximum (10RM) load over a total duration of 34 min. The exercises were performed in the following order: bench press, squat, T-bar row, and deadlift. Prior to the resistance exercise session, participants remained seated for 3 min to stabilize HR, during which, resting HR was recorded. To minimize cumulative muscular fatigue between exercises, a 2-min rest period was provided following each exercise. To standardize total exercise duration and movement tempo across participants, resistance exercises were performed at a controlled cadence of 2 s for the concentric phase and 2 s for the eccentric phase, resulting in a total contraction time of 40 s per set. Exercise cadence was regulated using a metronome. HR was recorded at three time points: immediately before exercise, immediately after exercise completion, and 1 min post-exercise to assess acute HR responses associated with resistance exercise (Figure 2).

### 2.3. Devices and Reference Measurements

Anthropometric measurements, including height and body mass, were obtained using a fixed automatic stadiometer and scale (BSM330, Biospace, Seoul, Republic of Korea). Body composition parameters, including body fat mass (BFM), percent body fat (PBF), and lean body mass (LBM), were assessed using a bioelectrical impedance analysis (BIA) device (InBody 720; Biospace, Seoul, Republic of Korea).

HR measurements were obtained using a standard ECG monitoring system (Q-Stress System, Quinton/Cardiac Science Corporation, Bothell, WA, USA) with disposable ECG electrodes (Kendall, Cardinal Health, Dublin, OH, USA), which served as the reference standard. ECG-derived HR was used to establish THR zones and to continuously monitor HR responses during endurance exercise. Smartwatch-derived HR measurements were simultaneously collected and compared using four commercially available devices: Apple Watch Series 7 (Apple Inc., Cupertino, CA, USA), Galaxy Watch 4 (Samsung Electronics, Seoul, Republic of Korea), Fitbit Charge 5 (Fitbit Inc., San Francisco, CA, USA), and Garmin Vivosmart 5 (Garmin Ltd., Schaffhausen, Switzerland).

EE was measured using a motor-driven treadmill (Quinton TM65; Quinton, Bothell, WA, USA) in conjunction with a metabolic measurement system (TrueOne 2400, ParvoMedics, Sandy, UT, USA), which served as the reference method based on indirect calorimetry. Smartwatch-derived EE values from the four devices (Apple, Galaxy, Fitbit, and Garmin) were obtained from the corresponding manufacturer-specific applications and compared with values derived from indirect calorimetry.

Prior to data collection, participants’ demographic and anthropometric information, including age, sex, height, and body mass, was entered into each smartwatch according to the manufacturers’ guidelines. All devices were initialized and configured individually for each participant before the experimental trials. EE estimation algorithms in wearable devices are known to be highly dependent on user-specific input parameters, such as age, sex, height, body mass, and body composition [18]. Variations in these inputs can directly influence the estimated EE values. To minimize variability associated with these factors, the same set of participant information was consistently applied across all devices. These input variables were carefully controlled to ensure comparability of EE measurements between devices.

### 2.4. Statistical Analyses

All statistical analyses were performed using SPSS Statistics version 27.0 (IBM Corp., Armonk, NY, USA). Changes in HR during endurance and resistance exercise were analyzed using a two-way repeated-measures analysis of variance (ANOVA), with device and measurement time as within-subject factors. Main effects of device and time, as well as their interaction effects, were examined. Post hoc comparisons were conducted using the Bonferroni correction. Differences in EE among devices were evaluated using a one-way repeated-measures ANOVA. The associations between smartwatch-derived measurements and reference-standard measurements (ECG for HR and indirect calorimetry for EE) during endurance and resistance exercise were assessed using Pearson correlation analysis. Agreement between smartwatch-derived measurements and reference methods was evaluated using intraclass correlation coefficients (ICCs) based on a two-way mixed-effects model with absolute agreement. Both single-measure and average-measure ICC values were reported. In addition, Bland–Altman analyses were performed to assess mean differences and 95% limits of agreement between each smartwatch and the corresponding reference method. All statistical tests were two-tailed, and the level of statistical significance was set at *p* < 0.05.

## 3. Results

### 3.1. Endurance Exercise Results

#### 3.1.1. HR Across Devices at Rest and During Endurance Exercise

**Figure 3:**
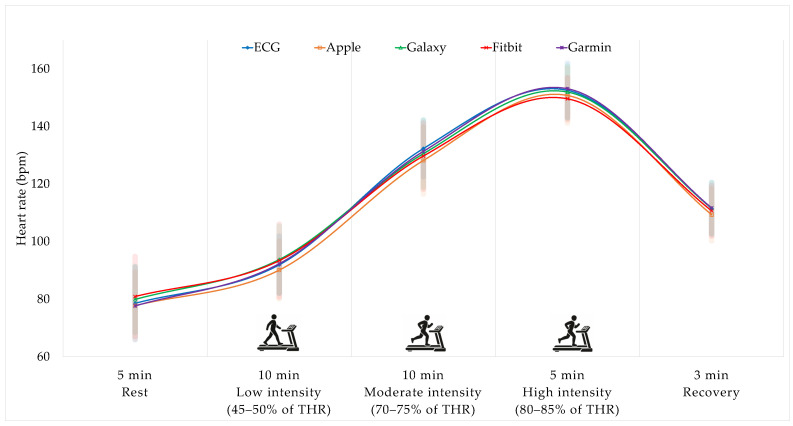
Changes in heart rate at rest and during endurance exercise across devices. Exercise intensity was prescribed using the Karvonen formula and consisted of low- (45–50% THR), moderate- (70–75% THR), and high-intensity (80–85% THR) stages, followed by recovery. Heart rate was recorded at rest and 30 s after reaching each intensity stage.

HR measurements obtained at rest and during endurance exercise using ECG and four smartwatches are presented in Figure 3. Two-way repeated-measures ANOVA revealed no significant main effect of device on HR (*F*(2.19, 133.46) = 1.09, *p* = 0.34, partial η^2^ = 0.018). In contrast, a significant main effect of time was observed (*F*(2.93, 178.46) = 1089.70, *p* < 0.001, partial η^2^ = 0.947), along with a significant device × time interaction (*F*(6.38, 389.07) = 4.07, *p* < 0.001, partial η^2^ = 0.063). Post hoc analyses indicated that HR increased progressively from rest to low-, moderate-, and high-intensity exercise across all devices (*p* < 0.05), followed by a significant decrease during the recovery period (*p* < 0.05).

#### 3.1.2. Correlation Analysis of HR During Endurance Exercise

Pearson correlation analysis demonstrated significant positive associations between ECG-derived HR and smartwatch-derived HR during endurance exercise for all devices (*p* < 0.001). Apple (*r* = 0.913) and Garmin (*r* = 0.897) exhibited very strong correlations with ECG, while Galaxy also demonstrated a high level of agreement (*r* = 0.881). In contrast, Fitbit showed a comparatively lower correlation with ECG (*r* = 0.637).

#### 3.1.3. ICC of HR During Endurance Exercise

Agreement between ECG-derived and smartwatch-derived mean HR values during endurance exercise was assessed using ICCs based on a two-way mixed-effects model with absolute agreement. The single-measure ICC was 0.847 (95% CI: 0.789–0.895, *p* < 0.001), indicating good reliability, whereas the average-measure ICC was 0.965 (95% CI: 0.949–0.977, *p* < 0.001), indicating excellent reliability.

#### 3.1.4. Bland–Altman Analysis of HR During Endurance Exercise

**Figure 4:**
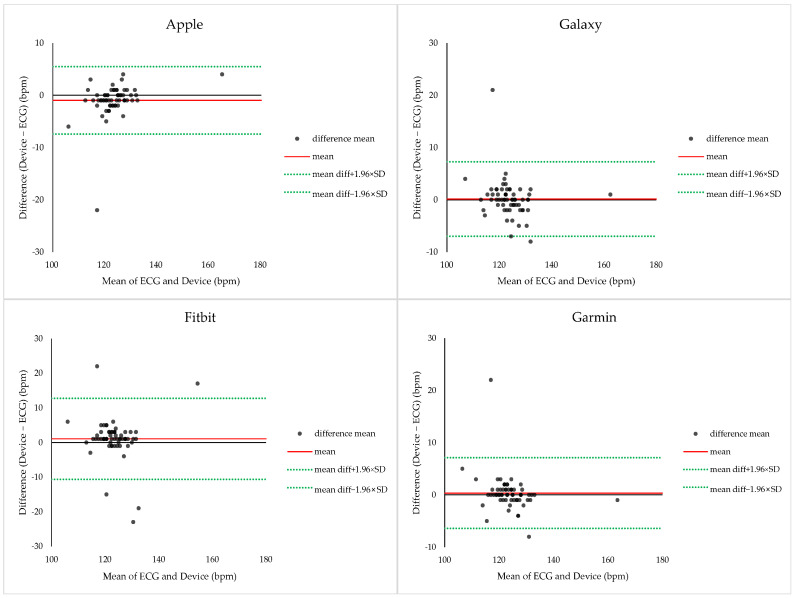
Bland–Altman plots comparing heart rate measurements obtained from ECG and smartwatches during endurance exercise. Each plot shows the mean heart rate derived from ECG and the smartwatch against the difference between the two measurements. The solid red line represents the mean bias, and the dashed blue lines indicate the 95% limits of agreement (LOA = bias ± 1.96 × SD).

Bland–Altman analysis was performed to evaluate agreement between ECG-derived HR and smartwatch-derived HR during endurance exercise (Figure 4). The mean bias for Apple was −0.97 ± 3.29 bpm, with 95% limits of agreement (LOA) ranging from −7.41 to +5.48 bpm. Galaxy showed a bias of +0.15 ± 3.63 bpm (−6.97 to +7.26 bpm), Fitbit showed a bias of +1.08 ± 5.97 bpm (−10.62 to +12.78 bpm), and Garmin showed a bias of +0.35 ± 3.45 bpm (−6.41 to +7.12 bpm). Across all devices, HR measurements were largely within ±10 bpm of ECG values, indicating a high level of agreement, with Apple and Galaxy demonstrating the narrowest limits of agreement.

#### 3.1.5. EE Across Devices During Endurance Exercise

EE values obtained during endurance exercise are presented in Table 1. One-way repeated-measures ANOVA revealed a significant main effect of device on EE (*F*(2.79, 170.32) = 59.99, *p* < 0.001, partial η^2^ = 0.496). Post hoc analyses indicated significant differences between indirect calorimetry and all smartwatch-derived EE values (*p* < 0.05).

#### 3.1.6. Correlation Analysis of EE During Endurance Exercise

Pearson correlation analysis revealed significant positive associations between EE measured by indirect calorimetry and smartwatch-derived EE during endurance exercise (*p* < 0.05). Correlation coefficients were highest for Apple (*r* = 0.591), followed by Galaxy (*r* = 0.487), Fitbit (*r* = 0.451), and Garmin (*r* = 0.262). However, all correlations were moderate to weak in magnitude.

#### 3.1.7. ICC of EE During Endurance Exercise

Agreement between smartwatch-derived EE and calorimetry-derived EE was evaluated using ICCs based on a two-way mixed-effects model with absolute agreement. The single-measure ICC was 0.377 (95% CI: 0.184–0.556, *p* < 0.001), indicating poor reliability. In contrast, the average-measure ICC was 0.752 (95% CI: 0.530–0.862, *p* < 0.001), indicating moderate-to-good reliability.

#### 3.1.8. Bland–Altman Analysis of EE During Endurance Exercise

**Figure 5:**
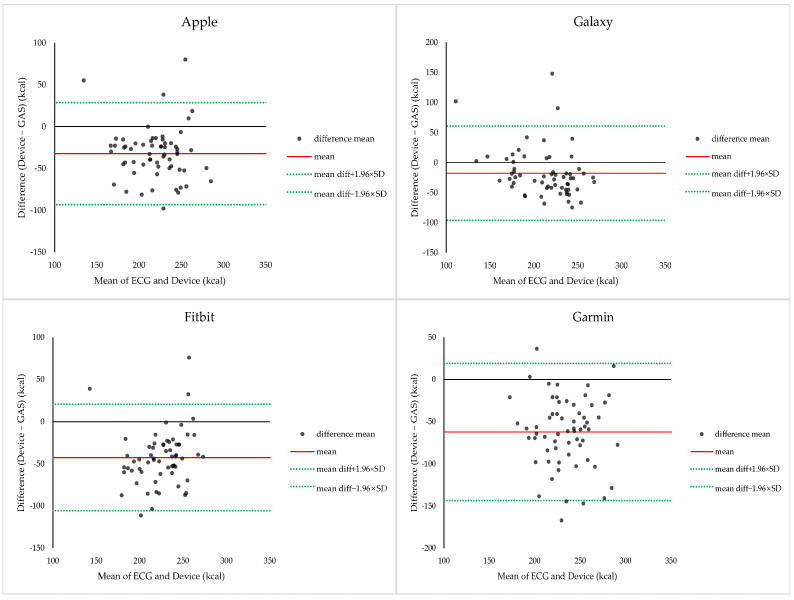
Bland–Altman plots comparing energy expenditure measurements obtained from indirect calorimetry and smartwatches during endurance exercise. Each plot displays the mean energy expenditure derived from indirect calorimetry and the smartwatch (*x*-axis) against the difference between the two measurements (*y*-axis). The solid red line represents the mean bias, and the dashed lines indicate the 95% limits of agreement (bias ± 1.96 × SD).

Bland–Altman analysis comparing smartwatch-derived EE and calorimetry-derived EE during endurance exercise is shown in Figure 5. Apple demonstrated a mean bias of −32.30 ± 31.11 kcal, with 95% LOA ranging from −93.27 to +28.68 kcal, indicating substantial underestimation. Galaxy (−17.75 ± 40.08 kcal; −96.31 to +60.81 kcal) and Garmin (−62.12 ± 41.47 kcal; −143.39 to +19.16 kcal) exhibited similar underestimation patterns. Fitbit also demonstrated underestimation (−42.44 kcal); however, the 95% LOA were wide (−105.59 to +20.71 kcal), indicating poor consistency.

### 3.2. Resistance Exercise Results

#### 3.2.1. HR Across Devices at Rest and During Resistance Exercise

**Figure 6:**
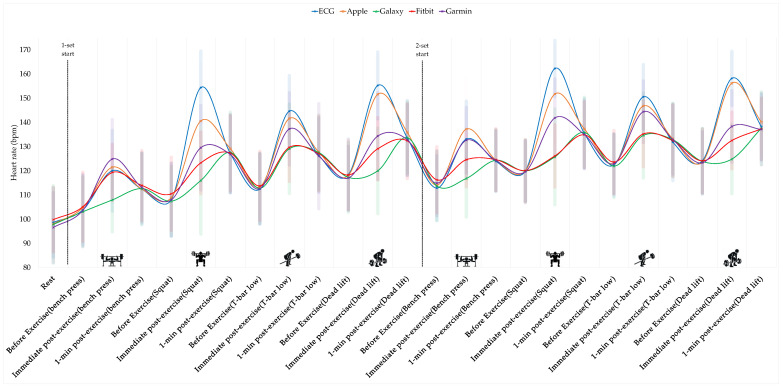
Changes in heart rate measured by ECG and commercial smartwatches during resistance exercise. Heart rate responses were recorded at rest, before exercise, immediately after exercise, and 1 min after exercise for each exercise set (bench press, squat, T-bar row, and deadlift). The dashed vertical line indicates the onset of the exercise sets. Lines represent mean values for ECG, Apple Watch, Galaxy Watch, Fitbit, and Garmin.

Changes in HR across devices at rest and during resistance exercise are presented in Figure 6. Two-way repeated-measures ANOVA revealed significant main effects of device (*F*(3.08, 187.62) = 155.14, *p* < 0.001, partial η^2^ = 0.718) and time (*F*(8.02, 489.00) = 225.61, *p* < 0.001, partial η^2^ = 0.787), as well as a significant device × time interaction (*F*(19.62, 1196.92) = 47.14, *p* < 0.001, partial η^2^ = 0.436). Post hoc analyses indicated significant differences in HR between ECG and three devices (Galaxy, Fitbit, and Garmin) (*p* < 0.05), whereas no significant difference was observed between ECG and Apple (*p* > 0.05). In addition, HR at rest was significantly lower than HR at all other measurement time points (*p* < 0.05).

#### 3.2.2. Correlation Analysis of HR During Resistance Exercise

Pearson correlation analysis demonstrated very strong positive associations between ECG-derived HR and smartwatch-derived HR during resistance exercise for all devices (*p* < 0.001). Specifically, correlation coefficients between ECG and Apple (*r* = 0.970), Galaxy (*r* = 0.959), Fitbit (*r* = 0.959), and Garmin (*r* = 0.960) indicated that all smartwatches closely tracked HR changes measured by ECG.

#### 3.2.3. ICC of HR During Resistance Exercise

Agreement between ECG-derived and smartwatch-derived mean HR values during resistance exercise was assessed using ICCs based on a two-way mixed-effects model with absolute agreement. The single-measure ICC was 0.949 (95% CI: 0.888–0.974, *p* < 0.001), indicating excellent reliability, while the average-measure ICC was 0.989 (95% CI: 0.975–0.995, *p* < 0.001), indicating near-perfect reliability.

#### 3.2.4. Bland–Altman Analysis of HR During Resistance Exercise

**Figure 7:**
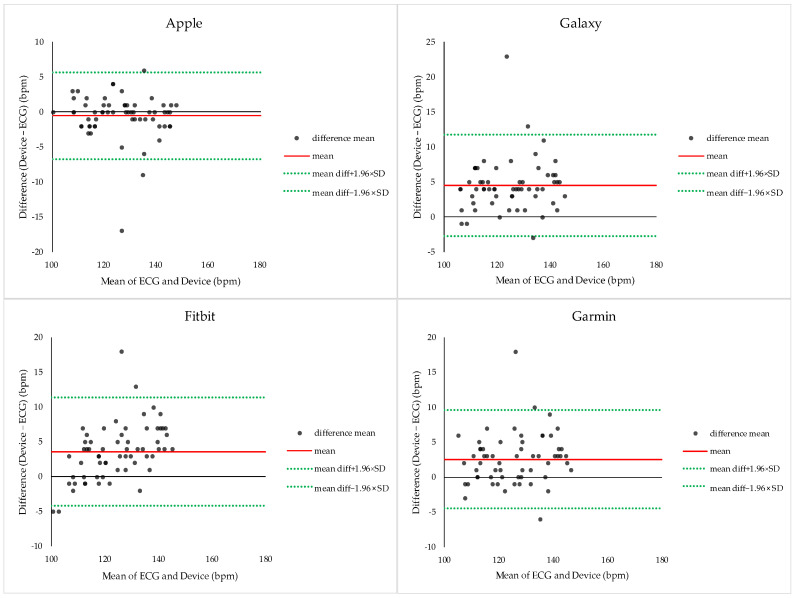
Bland–Altman plots comparing heart rate measurements obtained from ECG and smartwatches during resistance exercise. Each plot shows the mean heart rate derived from ECG and the smartwatch (*x*-axis) against the difference between the two measurements (*y*-axis). The solid red line represents the mean bias, and the dashed lines indicate the 95% limits of agreement (bias ± 1.96 × SD).

Bland–Altman analysis was conducted to evaluate agreement between ECG-derived and smartwatch-derived HR during resistance exercise (Figure 7). Apple demonstrated a mean bias of −0.53 ± 3.18 bpm, with 95% limits of agreement (LOA) ranging from −6.76 to +5.69 bpm. Galaxy showed a bias of +4.48 ± 3.70 bpm (−2.77 to +11.74 bpm), Fitbit showed a bias of +3.60 ± 3.97 bpm (−4.18 to +11.38 bpm), and Garmin showed a bias of +2.56 ± 3.62 bpm (−4.52 to +9.65 bpm). Across all devices, HR measurements exhibited relatively narrow error ranges compared with ECG, indicating strong agreement during resistance exercise.

#### 3.2.5. EE Across Devices During Resistance Exercise

EE values obtained during resistance exercise are presented in Table 2. One-way repeated-measures ANOVA revealed a significant main effect of device on EE (*F*(2.42, 147.66) = 215.44, *p* < 0.001, partial η^2^ = 0.779). Post hoc analyses indicated significant differences between indirect calorimetry and Apple, Galaxy, and Garmin (*p* < 0.05). In contrast, no significant difference was observed between indirect calorimetry and Fitbit (*p* > 0.05).

#### 3.2.6. Correlation Analysis of EE During Resistance Exercise

Pearson correlation analysis revealed significant positive associations between EE measured by indirect calorimetry and smartwatch-derived EE from Apple (*r* = 0.344) and Garmin (*r* = 0.321) (*p* < 0.05). However, no significant correlations were observed for Galaxy (*r* = 0.242) or Fitbit (*r* = 0.102) (*p* > 0.05). Overall, correlations between smartwatch-derived EE and calorimetry-derived EE were weak during resistance exercise.

#### 3.2.7. ICC of EE During Resistance Exercise

Agreement between smartwatch-derived EE and calorimetry-derived EE during resistance exercise was evaluated using ICCs based on a two-way mixed-effects model with absolute agreement. The single-measure ICC was 0.140 (95% CI: 0.022–0.295, *p* < 0.001), indicating very poor reliability. The average-measure ICC was 0.448 (95% CI: 0.102–0.676, *p* < 0.001), reflecting poor-to-fair reliability.

#### 3.2.8. Bland–Altman Analysis of EE During Resistance Exercise

**Figure 8:**
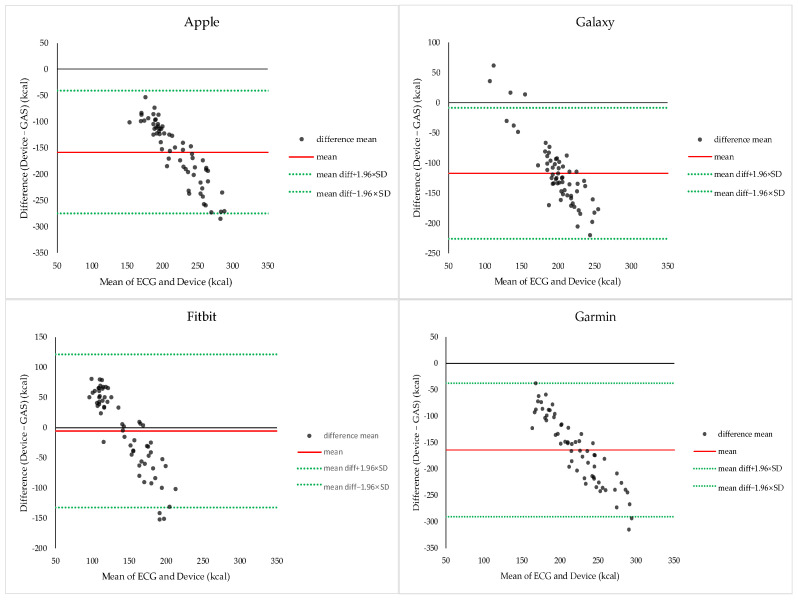
Bland–Altman plots comparing energy expenditure measurements obtained from indirect calorimetry and smartwatches during resistance exercise. Each plot shows the mean energy expenditure derived from indirect calorimetry and the smartwatch (*x*-axis) against the difference between the two measurements (*y*-axis). The solid red line represents the mean bias, and the dashed lines indicate the 95% limits of agreement (bias ± 1.96 × SD).

Bland–Altman analysis comparing smartwatch-derived EE and calorimetry-derived EE during resistance exercise is presented in Figure 8. The mean bias relative to indirect calorimetry was −158.30 kcal for Apple, −117.30 kcal for Galaxy, −5.16 kcal for Fitbit, and −163.92 kcal for Garmin. Apple, Galaxy, and Garmin consistently underestimated EE, whereas Fitbit showed a small mean bias but wide limits of agreement, indicating low measurement consistency.

## 4. Discussion

Smartwatches are representative wearable devices that provide real-time physiological information such as HR and EE during exercise. Owing to these capabilities, the use of smartwatches has rapidly expanded not only in the fitness industry, but also in the field of digital healthcare [1]. In practice, smartwatches are widely used to monitor health-related indicators, such as physical activity level, exercise intensity, and weight management [3,4,5], and their clinical applications, including cardiovascular disease management, are also increasing [14,15,19]. The HR measured by smartwatches is primarily estimated from PPG signals. However, wrist-worn PPG sensors are susceptible to motion artifacts caused by body movements. Therefore, most devices incorporate signals from inertial measurement units (IMUs) such as accelerometers and gyroscopes to quantify motion, reduce motion artifacts, and improve signal quality before estimating HR through algorithmic processing [20,21,22]. One possible explanation for the observed differences in HR accuracy between the exercise modalities may be related to the physiological characteristics of PPG-based measurements. PPG-derived HR estimation relies on detecting the temporal intervals between successive pulse wave peaks, which may be influenced by pulse wave velocity and peripheral vascular properties [23]. Previous studies have shown that PPG signals reflect changes in peripheral blood flow and vascular dynamics, including vascular responses associated with autonomic regulation, and that timing-related pulse features can vary with hemodynamic conditions [23,24]. Therefore, dynamic vascular changes during exercise may affect the morphology and timing of the PPG waveform, potentially contributing to variability in smartwatch-derived HR measurements. This physiological consideration may partly explain the modality-specific differences (endurance exercise vs. resistance exercise) observed in the present study.

In contrast, EE is typically estimated using algorithms that integrate HR signals, accelerometer data, and individual characteristics such as age and body mass. Although mean EE values appeared higher in some devices, Bland–Altman analysis revealed a proportional bias, indicating systematic underestimation across exercise intensity levels. Previous studies have reported that such estimates show moderate levels of accuracy with considerable variability depending on the device and type of activity performed [13,25].

The present study demonstrated that the accuracy of smartwatch-based HR measurements during endurance exercise is very high. HRs showed clear changes across exercise stages, exhibiting a typical cardiovascular response in which HRs increased progressively from rest to low-, moderate-, and high-intensity exercise and decreased during the recovery period across all devices. In contrast, no significant differences were observed between the devices, indicating that the average HR values measured by the smartwatches were comparable to those obtained from the ECG during endurance exercise. These findings suggest that PPG-based smartwatches can achieve a high level of HR measurement validity that is comparable to that of ECG in endurance exercise environments, where peripheral blood flow remains relatively stable and rhythmic movements are maintained. Although a statistically significant interaction between the device and exercise stage was observed, the magnitude of this effect was relatively small, suggesting that minor differences in HR tracking may occur during changes in exercise intensity without reflecting clinically meaningful discrepancies in absolute HR values.

Furthermore, strong correlations with ECG values were observed for all devices (*r* = 0.64–0.91). These findings may be explained by the relatively regular HR responses and stable peripheral blood flow that occur during endurance exercise, which are the conditions under which PPG-based HR monitoring can maintain high signal quality. In addition, endurance exercises involve repetitive and periodic movements, which likely produce consistent accelerometer signals and facilitate the effective correction of motion artifacts. Previous studies have also reported relatively high HR measurement accuracy for devices such as Apple, Garmin, and Galaxy during endurance-type activities, such as walking and running [26,27,28], and the present findings are consistent with these reports. The relatively lower accuracy observed for the Fitbit in certain segments may be partly related to differences in signal processing and smoothing algorithms designed to stabilize wrist-based PPG signals. Although such algorithms can effectively reduce noise, they may introduce temporal delays or attenuate rapid HR changes during periods of sudden exercise onset or transitions in exercise intensity [12,22,29,30].

In the present study, the Fitbit device tended to overestimate the HR at lower values and underestimate it at higher values, particularly during transitions in exercise intensity. This pattern is indicative of a proportional bias, which has been previously reported for consumer wearable devices. Similar findings were reported by Nelson and Allen [30], who demonstrated that the Fitbit Charge 2 exhibited overestimation at lower HRs and underestimation at higher HRs during dynamic physical activity. However, the present findings suggest that this bias becomes more pronounced during rapid transitions in exercise intensity, indicating a potential limitation in the responsiveness of the device to dynamic physiological changes.

Interestingly, although the present study utilized a later-generation device (Fitbit Charge 5), the pattern of proportional bias observed was consistent with that reported for earlier models, such as the Fitbit Charge 2 [30]. This finding suggests that certain characteristics of HR estimation algorithms in Fitbit devices remain relatively consistent across device generations. One possible explanation for this phenomenon is that wearable devices may apply smoothing or filtering algorithms to reduce the signal noise and motion artifacts. While such approaches can improve signal stability under steady-state conditions, they may attenuate rapid fluctuations in the HR, resulting in delayed or dampened responses during periods of increasing or decreasing exercise intensity. Therefore, the persistence of similar bias patterns across device generations may reflect the inherent characteristics of the underlying signal processing strategies rather than the limitations of a specific device model.

These findings further suggest that signal-processing characteristics such as smoothing and motion artifact correction may contribute to the observed bias patterns. Although such approaches are effective in improving signal stability, they may also attenuate rapid HR fluctuations, particularly during exercise intensity transitions.

In addition to the observed proportional bias, the Bland–Altman analysis revealed notable differences in measurement precision across devices. Specifically, during endurance exercise, the Fitbit device exhibited considerably wider limits of agreement than Apple, Galaxy, and Garmin, indicating lower consistency in HR measurements. This discrepancy may be related to the differences in signal processing strategies across devices. In particular, stronger smoothing or filtering algorithms applied to reduce motion artifacts may increase variability in the estimated HR values under dynamic conditions [12,22]. Furthermore, differences in sensor performance, sampling frequency, and sensor fusion techniques may also contribute to the observed variability [22].

Therefore, proportional bias in wearable devices should not only be considered as a general limitation but also as a device-specific characteristic that may vary depending on the exercise conditions and algorithmic design. In addition, the present results highlight the potential value of conducting stratified Bland–Altman analyses across different HR ranges to better characterize the proportional bias in wearable devices. This approach may provide further insights into device-specific performance under varying physiological conditions and should be considered in future studies. However, further studies that directly compare multiple device generations under controlled conditions are required to confirm this interpretation.

In contrast, the EE estimation during endurance exercise showed substantial discrepancies across all smartwatch devices when compared with indirect calorimetry. Bland–Altman analysis indicated that Apple, Galaxy, and Garmin systematically underestimated EE by approximately −17 to −62 kcal relative to indirect calorimetry, whereas Fitbit showed a smaller mean bias but wider limits of agreement, indicating lower measurement consistency. These discrepancies may be explained by the fact that smartwatches do not directly measure oxygen uptake but instead estimate EE using HR-based regression models or algorithms that incorporate user profile variables, such as age, body mass, and sex [31,32,33]. Moreover, the relationship between HR and oxygen uptake becomes increasingly nonlinear at higher exercise intensities, limiting the ability of HR-based models to accurately predict metabolic energy expenditure. These findings are consistent with those of previous studies reporting the limited accuracy of smartwatch-based EE estimations.

Interestingly, smartwatch-based HR measurements also maintained relatively high accuracy during the resistance exercise. In the present study, HR correlations exceeded r > 0.95, and ICC values ranged from 0.94 to 0.99, indicating excellent agreement. Bland–Altman analysis further showed that the mean bias remained within ±4 bpm. These results suggest that PPG-based HR monitoring can remain relatively stable even during resistance exercise involving repeated muscle contractions and inter-set recovery periods. This may be partially explained by the movement characteristics of resistance exercises. Although transient trunk movements may occur, the wrist often remains relatively stable while gripping the exercise equipment, resulting in less continuous arm movement compared with endurance exercise and allowing wrist-based PPG signals to remain relatively stable [21,22,26,29].

However, the differences in HR measurements among the devices were more pronounced during resistance exercise. Notably, the Apple Watch demonstrated a closer alignment with ECG values in capturing peak HR values than the other devices, suggesting a greater ability to track rapid transient changes in HR. This may indicate that certain device-specific algorithms or sensor-fusion strategies enable a more responsive detection of dynamic physiological signals.

In contrast, the Galaxy, Fitbit, and Garmin devices exhibited a temporal lag in HR measurements relative to the ECG reference, particularly during resistance exercise involving rapid fluctuations in HR. This delay may be attributed to the signal smoothing or filtering processes designed to reduce noise and motion artifacts [24]. Although such approaches can improve signal stability, they may also introduce latency to reflect rapid physiological changes. These findings suggest that differences in temporal responsiveness among devices may influence the accuracy of peak HR detection and exercise intensity monitoring, particularly under conditions characterized by intermittent contractions and rapidly changing cardiovascular demands. Post hoc analyses indicated that the Apple Watch showed no significant difference from the ECG values, whereas the Galaxy, Fitbit, and Garmin devices demonstrated significant differences compared to the ECG values. This finding suggests that the accuracy of PPG-based HR measurements during resistance exercise varies across devices. During resistance exercise, repeated muscle contractions, isometric tension, vascular compression, and motion artifacts may degrade the PPG signal quality. Under such conditions, differences in signal-processing algorithms or sensor-fusion strategies among devices may influence the HR measurement accuracy. To date, most wearable HR validation studies have focused primarily on endurance exercise [34], and the systematic validation of smartwatch HR measurement accuracy during resistance exercise using ECG as the reference standard remains limited. In this context, this study provides meaningful evidence by directly comparing multiple smartwatches with ECG during resistance exercise conditions that reflect realistic training environments.

EE estimation during resistance exercise showed very low accuracy across all devices. Apple, Galaxy, and Garmin consistently underestimated EE, whereas Fitbit showed a relatively small mean bias but wide limits of agreement, indicating poor measurement consistency. These findings may be explained by the physiological characteristics of resistance exercise. Resistance exercise is characterized by intermittent patterns involving short bouts of activity, followed by longer rest periods [35,36], localized muscle activation [36], and nonlinear relationships between HR and oxygen uptake [37,38]. Under these conditions, HR-based algorithms may not accurately reflect metabolic energy expenditure, and errors may accumulate when HR signals are converted into EE estimates. Previous studies have also reported a particularly low EE estimation accuracy of wearable devices during resistance exercises [11,12,13,38], and the present findings further confirm these limitations.

Overall, the present study demonstrates that smartwatch-based HR measurements show high validity during both endurance and resistance exercises, whereas EE estimation shows limited accuracy across both exercise modalities and particularly low reliability during resistance exercise. These findings suggest that smartwatches can be effectively used as HR-based exercise-monitoring tools; however, caution should be exercised when interpreting the calorie expenditure or metabolic estimates derived from such devices.

Importantly, these findings also have practical implications for real-world applications of wearable exercise monitoring technologies. Smartwatches are increasingly used for exercise intensity management, weight control, and integration with digital health platforms, and many users rely on physiological indicators provided by these devices to guide their exercise or health management decisions. However, the present results indicate that, while HR-based metrics may be relatively reliable, EE estimates may contain substantial errors depending on the type of exercise performed. In particular, during resistance exercise characterized by intermittent contractions and localized muscle activation, smartwatch-based EE estimation may fail to adequately reflect the actual metabolic expenditure. Therefore, future advancements in wearable exercise monitoring technologies may benefit from sensor-fusion approaches that integrate additional physiological signals, such as electromyography (EMG), muscle activation patterns, or other multimodal biosignals, along with HR and accelerometer data. Furthermore, validation studies incorporating a wider range of exercise modalities and real-world exercise conditions may further expand the practical applicability of the physiological data obtained from wearable devices.

This study has several limitations that warrant consideration. First, the sample consisted exclusively of healthy adult men, limiting the generalizability to women, older adults, and clinical populations. Second, although the resistance exercise protocol reflected common training practices, it did not encompass the full range of resistance exercise variables, such as load progression or contraction velocity. Third, the present analysis focused on absolute EE accuracy and did not assess the reproducibility or relative changes across repeated sessions. Finally, the device placement and strap tightness were standardized and may not reflect real-world usage conditions. Addressing these limitations in future studies is essential for advancing the validity and applicability of smartwatch-derived physiological metrics.

## 5. Conclusions

This study systematically evaluated the accuracy of HR and EE measurements obtained using commercially available smartwatches during endurance and resistance exercises. The results demonstrate that smartwatch-derived HR measurements exhibit very high accuracy regardless of the exercise modality, showing strong correlations and excellent agreement with ECG. These findings support the use of smartwatches as reliable tools for exercise intensity monitoring and HR-based training prescriptions across diverse exercise contexts.

However, device-specific differences were observed under dynamic exercise conditions. In particular, certain devices exhibited proportional bias, characterized by overestimation at lower HR values and underestimation at higher HR values, as well as a temporal lag in tracking rapid HR fluctuations. These findings suggest that algorithmic characteristics and signal-processing strategies may influence HR measurement performance, particularly during transitions in exercise intensity and resistance.

In contrast, smartwatch-derived EE estimates failed to accurately reflect the actual metabolic demand during both endurance and resistance exercises, with particularly pronounced bias and poor agreement observed during the resistance exercise. This limitation likely stems from the reliance on proprietary, HR- and motion-based algorithms that do not adequately capture the physiological characteristics of intermittent exercise patterns involving short work bouts and prolonged rest periods. Furthermore, under conditions involving high-exercise-intensity or nonuniform movement patterns, the relationship between HR and metabolic demand may become nonlinear, potentially amplifying estimation errors when EE is inferred primarily from HR signals.

Accordingly, the EE values provided by smartwatches should be interpreted with caution and may be more appropriately used to assess relative changes in activity or overall movement trends rather than as absolute indicators of energy expenditure. Overall, this study confirms the robustness of smartwatch-based HR monitoring while clearly delineating the limitations of EE estimation across exercise modalities. Future research should address these limitations by incorporating diverse populations, resistance exercise protocols, and advanced multimodal biosignal approaches to improve the practical applicability of smartwatch-derived physiological metrics.

## Figures and Tables

**Table 1:** Energy expenditure measured by indirect calorimetry and smartwatches during endurance exercise.

Device	Energy Expenditure (kcal)
Gas analyzer	203.75 ± 32.99 ^a^
Apple	232.87 ± 35.61 ^b^
Galaxy	221.50 ± 43.92 ^c^
Fitbit	246.19 ± 27.98 ^d^
Garmin	265.87 ± 35.23 ^e^

Values are presented as mean ± standard deviation. Different superscript letters indicate significant differences among devices (*p* < 0.05).

**Table 2:** Energy expenditure measured by indirect calorimetry and smartwatches during resistance exercise.

Device	Energy Expenditure (kcal)
Gas analyzer	140.79 ± 14.37 ^a^
Apple	299.10 ± 63.14 ^b^
Galaxy	258.10 ± 57.27 ^c^
Fitbit	145.95 ± 64.67 ^a^
Garmin	304.71 ± 67.66 ^d^

Values are presented as mean ± standard deviation. Different superscript letters indicate significant differences among devices (*p* < 0.05).

## Data Availability

The data presented in this study are available from the corresponding author upon reasonable request.

## References

[1] American College of Sports Medicine (ACSM) Wearable Technology Named Top Fitness Trend for 2024.

[2] Mardonova M., Choi Y. (2018). Review of Wearable Device Technology and Its Applications to the Mining Industry. Energies.

[3] Jin W., Deng W. (2025). Research on the design of smartwatch health information visualization presentation under different motion scenarios. Sci. Rep..

[4] Kashyap R., Theraiyan E. (2025). A Study on Impact of Smart Watches Utilization in Present Young Generation with Special Reference in Chennai Region. Int. J. Res. Anal. Rev..

[5] Liu Y., Zhang H., Wang J., Li X. (2025). Validity of four low-cost smartwatches in estimating energy expenditure during cycling in Chinese untrained women. Front. Physiol..

[6] Hertzman A.B. (1938). The blood supply of various skin areas as estimated by the photoelectric plethysmograph. Am. J. Physiol. Legacy Content.

[7] Maeda Y., Sekine M., Tamura T. (2011). The Advantages of Wearable Green Reflected Photoplethysmography. J. Med. Syst..

[8] Raja J.M., Elsakr C., Roman S., Cave B., Pour-Ghaz I., Nanda A., Maturana M., Khouzam R.N. (2019). Apple Watch, wearables, and heart rhythm: Where do we stand?. Ann. Transl. Med..

[9] Butler M.J., Crowe J.A., Hayes-Gill B.R., Rodmell P.I. (2016). Motion limitations of non-contact photoplethysmography due to the optical and topological properties of skin. Physiol. Meas..

[10] Koerber D., Khan S., Shamsheri T., Kirubarajan A., Mehta S. (2022). The effect of skin tone on accuracy of heart rate measurement in wearable devices: A systematic review. J. Am. Coll. Cardiol..

[11] Boudreaux B.D., Hebert E.P., Hollander D.B., Williams B.M., Cormier C.L., Naquin M.R., Gillan W.W., Gusew E.E., Kraemer R.R. (2018). Validity of wearable activity monitors during cycling and resistance exercise. Med. Sci. Sports Exerc..

[12] Shcherbina A., Mattsson C.M., Waggott D., Salisbury H., Christle J.W., Hastie T., Wheeler M.T., Ashley E.A. (2017). Accuracy in wrist-worn, sensor-based measurements of heart rate and energy expenditure. J. Pers. Med..

[13] Fuller D., Colwell E., Low J., Orychock K., Tobin M.A., Simango B., Buote R., Van Heerden D., Luan H., Cullen K. (2020). Reliability and validity of commercially available wearable devices for measuring steps, energy expenditure, and heart rate: Systematic Review. JMIR mHealth uHealth.

[14] Adepoju V.A. (2024). Wearable technology in the management of chronic diseases. Cardiovasc. Digit. Health J..

[15] Jamieson A., Turakhia M.P., Desai M. (2025). A guide to consumer-grade wearables in cardiovascular care. Nat. Rev. Cardiol..

[16] Karvonen M.J., Kentala E., Mustala O. (1957). The effects of training on heart rate: A longitudinal study. Ann. Med. Exp. Biol. Fenn..

[17] Robergs R.A., Landwehr R. (2002). The surprising history of the “HRmax = 220 − age” equation. J. Exerc. Physiol..

[18] Kim M.-S., Seong J.-H. (2025). A Personalized Energy Expenditure Estimation Method Using Modified MET and Heart Rate-Based DQN. Sensors.

[19] Jang M.K., Cho Y.K., Moon J.Y., Min S.H., Hwang J.H., Jung C.H. (2026). Impact of smart watch mobile application on the risk treatment of type 2 diabetes mellitus (iSMART-DM). Prim. Care Diabetes.

[20] Hao Z., Wang J., Zhang G., Gao L., Zhang X., Liu J., Zhang X., Yang X., Lai Z. (2024). PPG heart rate extraction algorithm based on motion artifact intensity classification and removal framework. Biomed. Signal Process. Control.

[21] Lee H., Chung H., Ko H., Parisi A., Busacca A., Faes L., Pernice R., Lee J. (2022). Adaptive scheduling of acceleration and gyroscope for motion artifact cancellation in photoplethysmography. Comput. Methods Programs Biomed..

[22] Castaneda D., Esparza A., Ghamari M., Soltanpur C., Nazeran H. (2018). A review on wearable photoplethysmography sensors and their potential future applications in health care. Int. J. Biosens. Bioelectron..

[23] Allen J. (2007). Photoplethysmography and its application in clinical physiological measurement. Physiol. Meas..

[24] Tamura T., Maeda Y., Sekine M., Yoshida M. (2014). Wearable Photoplethysmographic Sensors—Past and Present. Electronics.

[25] Passler S., Bohrer J., Blöchinger L., Senner V. (2019). Validity of Wrist-Worn Activity Trackers for Estimating VO2max and Energy Expenditure. Int. J. Environ. Res. Public Health.

[26] Khushhal A., Nichols S., Evans W., Gleadall-Siddall D.O., Page R., O’Doherty A.F., Carroll S., Ingle L., Abt G. (2017). Validity and reliability of the Apple Watch for measuring heart rate during exercise. Sports Med. Int. Open.

[27] Pasadyn S., Soudan M., Gillinov M., Houghtaling P., Phelan D., Gillinov N., Bittel B., Desai M. (2019). Accuracy of commercially available heart rate monitors in athletes: A prospective study. Cardiovasc. Diagn. Ther..

[28] Nissen M., Slim S., Jäger K., Flaucher M., Huebner H., Danzberger N., Fasching P.A., Beckmann M.W., Gradl S., Eskofier B.M. (2022). Heart rate measurement accuracy of Fitbit Charge 4 and Samsung Galaxy Watch Active2: Device evaluation study. JMIR Form. Res..

[29] Zhang Z., Pi Z., Liu B. (2015). TROIKA: A general framework for heart rate monitoring using wrist-type photoplethysmographic signals during intensive physical exercise. IEEE Trans. Biomed. Eng..

[30] Nelson B.W., Allen N.B. (2019). Accuracy of consumer wearable heart rate measurement: Comparison of Apple Watch 3 and Fitbit Charge 2 with ambulatory ECG. JMIR mHealth uHealth.

[31] Seo Y., Lee Y., Lee D.T. (2025). Comparing heart rate and heart rate reserve for energy expenditure regression modeling during treadmill exercise. Int. J. Environ. Res. Public Health.

[32] O’Driscoll R., Turicchi J., Hopkins M., Horgan G.W., Finlayson G., Stubbs J.R. (2020). Improving energy expenditure estimates from wearable devices: A machine learning approach. J. Sports Sci..

[33] Le S., Wang X., Zhang T., Lei S.M., Cheng S., Yao W., Schumann M. (2022). Validity of Three Smartwatches in Estimating Energy Expenditure during Outdoor Walking and Running. Front. Physiol..

[34] Van Oost C.N., Masci F., Malisse A., Schyvens A.-M., Peters B., Dirix H., Ross V., Wets G., Neven A., Verbraecken J. (2025). Accuracy of Heart Rate Measurement Under Transient States: A Validation Study of Wearables for Real-Life Monitoring. Sensors.

[35] Bot S.D.M., Hollander A.P. (2000). The relationship between heart rate and oxygen uptake during non-steady state exercise. Ergonomics.

[36] Mitchell L., Wilson L., Duthie G., Pumpa K., Weakley J., Scott C., Slater G. (2024). Methods to Assess Energy Expenditure of Resistance Exercise: A Systematic Scoping Review. Sports Med..

[37] Reis V.M., Vianna J.M., Barbosa T.M., Garrido N., Vilaça Alves J., Carneiro A.L., Aidar F.J., Novaes J. (2019). Are Wearable Heart Rate Measurements Accurate to Estimate Aerobic Energy Cost during Low-Intensity Resistance Exercise?. PLoS ONE.

[38] O’Driscoll R., Turicchi J., Beaulieu K., Scott S., Matu J., Deighton K., Finlayson G., Stubbs J. (2020). How well do activity monitors estimate energy expenditure? A systematic review and meta-analysis. Br. J. Sports Med..

